# Plastid genes in motion: mechanisms and physiological implications of endosymbiotic gene transfer

**DOI:** 10.1093/jxb/erag086

**Published:** 2026-02-16

**Authors:** Yuyang Zhong, Femke Kuijl-van den Berg, Kin Pan Chung

**Affiliations:** Laboratory of Plant Physiology, Wageningen University & Research, Wageningen 6708 PB, the Netherlands; Laboratory of Plant Molecular Genetics, Graduate School of Agricultural and Life Sciences, The University of Tokyo, Tokyo 113-8657, Japan; Laboratory of Plant Physiology, Wageningen University & Research, Wageningen 6708 PB, the Netherlands; Laboratory of Plant Physiology, Wageningen University & Research, Wageningen 6708 PB, the Netherlands; Instituto de Hortofruticultura Subtropical y Mediterranea, Spain

**Keywords:** DNA repair, endosymbiotic gene transfer, evolution, genome editing, mitochondrial genome, NUMT, NUPT, plastidgenome

## Abstract

Recent research breakthroughs in plastid-to-nucleus gene transfer highlight its physiological importance as an endosymbiotic gene transfer process.

**Endosymbiotic gene transfer (EGT), the movement of DNA from endosymbionts to the host nuclear genome, represents one of the driving forces in eukaryotic evolution. Throughout the process of endosymbiosis, EGT profoundly shaped host–symbiont interactions and the evolution of nuclear genomes. Recent studies reveal that EGT remains an active process in plants, indicating that it is far from a relic of early eukaryotic evolution. Here, we focus on one type of EGT: plastid-to-nucleus gene transfer, and highlight latest research breakthroughs that shed light on its physiological significance and the molecular mechanisms underlying this ancient, yet ongoing dialogue between genomes.**

## A mosaic nuclear genome shaped by nuclear integrants of plastid DNA

The onset of primary plastid endosymbiosis set the stage for relentless gene transfer from the cyanobacterial endosymbiont to the host nucleus, progressively expanding the nuclear genome through the integration of cyanobacterium-derived DNA (Timmis *et al*., 2004). Remarkably, these gene transfer events have continued long after plastids became permanent organelles (Bock, 2017). Recent advances in genome sequencing and assembly have uncovered the widespread presence of nuclear integrants of plastid DNA (NUPTs) across diverse plant species. In *Arabidopsis thaliana*, approximately 18% of nuclear genes are of cyanobacterial origin (Martin *et al*., 2002). Moreover, large fragments of plastid DNA have been detected in the nuclear genomes of maize, rice, soybean, sorghum, and grapevine (Michalovová *et al*., 2013). Collectively, these findings highlight the mosaic nature of plant nuclear genomes decorated by NUPTs.

Since NUPTs affect not only the composition but also the overall architecture of the nuclear genome, extensive research has focused on their evolutionary fate and physiological impact. While some NUPTs are unstable and eventually eliminated (Sheppard and Timmis, 2009), others are retained and undergo various modifications, including mutation (Huang *et al*., 2005), fragmentation (Matsuo *et al*., 2005), and rearrangement (Huang *et al*., 2004). Epigenetic modification of NUPTs has also been reported, with some NUPTs becoming hypermethylated soon after integration. This hypermethylation is thought to represent a defense mechanism that protects the nuclear genome from foreign DNA (Yoshida *et al*., 2019).

## New homes, new roles

**Fig. 1.**
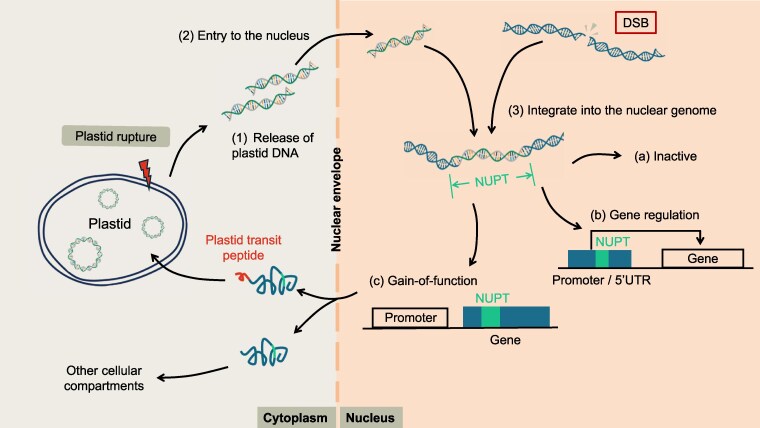
Formation and fate of NUPTs. Upon plastid rupture, plastid DNA may be released into the cytoplasm and subsequently enters the nucleus. Once inside the nucleus, plastid-derived DNA can integrate into the nuclear genome at sites of double-strand breaks (DSBs, indicated in the red box). Stable integration results in the formation of NUPTs, which can lead to several possible outcomes: (a) the NUPT remains transcriptionally inactive; (b) it integrates into regulatory regions, affecting the expression of neighboring genes; or (c) it acquires eukaryotic transcriptional elements, enabling it to function as a nuclear gene. In the latter case, the NUPT may either be targeted back to the plastid via a transit peptide, or be redirected to other cellular compartments to perform new functions.

Despite their potentially deleterious effects, NUPTs can serve as sources of genetic raw material, contributing to genomic diversity and innovation. Functional gene transfer can give rise to new nuclear genes in which NUPT-derived sequences encode proteins that are either targeted back to the plastid via a transit peptide to perform their ancestral functions, or redirected to other cellular compartments where they acquire new roles (Kleine *et al*., 2009). Notably, NUPTs can also modulate gene expression by acting as new regulatory elements (Yang *et al*., 2025) and remodel existing nuclear genes by contributing novel exon sequences (Noutsos *et al*., 2007) (Fig. 1).

A recent study further illustrated the physiological significance of such exon contributions from NUPTs. *BOOSTER* (*BSTR*), an orphan gene identified in *Populus trichocarpa* that lacks homologues in other lineages, comprises two exons of apparent endophytic origin, and a third exon derived from NUPTs. This third exon shares 94% sequence identity with the C-terminal region of the plastid-encoded Rubisco large subunit (*rbcL*) gene (Feyissa *et al*., 2025). BSTR is proposed to function as a nuclear-localized transcriptional repressor involved in nucleus-to-plastid anterograde signaling. BSTR enhances non-photochemical quenching responses, and its overexpression leads to improved photosynthetic efficiency and increased biomass accumulation (Feyissa *et al*., 2025). These findings indicate that even small NUPT-derived sequences (such as a portion of *rbcL*) can contribute to the evolution of novel nuclear genes with important physiological functions.

## The journey of plastid genes: from transfer to integration in the nuclear genome

Beyond their physiological implications, a central question in the field concerns how NUPTs originate. It is generally accepted that plastid-to-nucleus gene transfer proceeds through three key steps: (i) the release of plastid DNA from the organelle, (ii) its translocation to the nucleus, and (iii) its eventual integration into the nuclear genome (Fig. 1) (Bock and Timmis, 2008). However, the molecular mechanisms underlying these steps remain elusive.

The formation of NUPTs requires plastid DNA to be released from the organelle. Previous studies have shown that heat stress can compromise plastid membrane integrity, resulting in plastid rupture and the release of plastid DNA into the cytoplasm, from where it can ultimately reach the nucleus (Wang *et al*., 2012). Moreover, a high frequency of plastid-to-nucleus gene transfer has been observed in the male germline (Sheppard *et al*., 2008). During pollen maturation, plastids and their DNA undergo degradation (Chung, 2025), a process that could facilitate the release of plastid DNA into the cytoplasm. Interestingly, this release does not appear to be the rate-limiting step in plastid-to-nucleus gene transfer, as the frequency of plastid DNA translocation to the nucleus far exceeds that of successful integration (Sheppard *et al*., 2008). This suggests that the integration step represents the primary bottleneck in the establishment of NUPTs.

What, then, determines integration efficiency? For translocated plastid DNA to become stably incorporated into the nuclear genome, double-strand breaks (DSBs) in the nuclear genome provide potential entry points for integration (Wang *et al*., 2018). Thus, the cellular processes that regulate the abundance of DSBs likely represent key determinants of plastid DNA integration efficiency (Fig. 1). A recent study has provided important mechanistic insights into this critical integration step (Gonzalez-Duran *et al*., 2025). In plants, non-homologous end joining (NHEJ) and microhomology-mediated end joining (MMEJ) are major pathways responsible for DSB repairs in somatic cells (Miller *et al*., 2021). Impairment of these pathways increases the number of available DSB sites for plastid DNA integration. Consistent with this notion, *Nicotiana tabacum* mutants *lig4* (NHEJ-deficient) and *polq* (polymerase θ–mediated MMEJ-deficient) display a marked increase in plastid-to-nucleus gene transfer frequency (Gonzalez-Duran *et al*., 2025). These findings highlight that DNA repair pathways and nuclear genome accessibility are key determinants of formation of NUPTs.

## Open questions and future perspectives

While recent studies have greatly deepened our understanding of the physiological implications and molecular mechanisms underlying plastid-to-nucleus gene transfer, they have also raised further fundamental questions that extend to mitochondria-to-nucleus gene transfer and nuclear integrants of mitochondrial DNA (NUMTs).

### Endosymbiotic gene transfer and nuclear genome surveillance mechanisms

Frequent EGT events could act as a potent mutagenic force, driving the accumulation of NUPTs and NUMTs and thereby posing a serious threat to nuclear genome stability (Gonzalez-Duran *et al*., 2025). The DSB-repair machinery likely serves as a first line of defense, limiting opportunities for organellar DNA integration into the nuclear genome. As a second layer of protection, epigenetic modification and gene silencing mechanisms ensure that the majority of NUPTs and NUMTs remain transcriptionally inactive (Fig. 1). Collectively, these multilayered defense strategies suggest that the host cell has developed mechanisms (which may not have originally evolved to counteract EGT) which effectively protect the nuclear genome from excessive organellar DNA intrusion. Intriguingly, despite these safeguard mechanisms, the nuclear genome is not completely insulated from organellar DNA insertions. Since DSB-repair and epigenetic regulation activities vary with developmental stage and cellular state (Boyko *et al*., 2006; Hemenway and Gehring, 2023), the effectiveness of these defense mechanisms, and thus the extent and impact of EGT, may differ accordingly. Deciphering how these cellular processes are coordinated will be key to understanding the evolutionary dynamics that continue to shape nuclear genomes today.

### Functional endosymbiotic gene transfer and organellar gene loss

An intriguing consequence of EGT is that once an organellar gene has been successfully relocated to the nucleus and functionally expressed there, the original organellar gene becomes redundant. This redundancy can lead to pseudogenization of the organellar gene, ultimately rendering it non-functional. As the nuclear-encoded version becomes essential, the gene replacement becomes irreversible. Over evolutionary timescales, this process may result in the eventual loss of the gene from the organellar genome (Selosse *et al*., 2001). Recent research provides compelling support for this evolutionary trajectory. In Arabidopsis, the function of the plastid gene *RPS16* has been substituted by its nuclear-encoded counterpart (Roy *et al*., 2010). By using a reverse genetic approach, it has been shown that the plastid-encoded *RPS16* is a transcribed pseudogene (Ruf *et al*., 2025), suggesting that pseudogenization represents an intermediate step toward plastid gene loss. Notably, in species such as tomato and rice, *RPS16* is also expressed from the nuclear genome (Ueda *et al*., 2008), although it remains unclear whether their plastid *RPS16* copies have undergone pseudogenization. It is conceivable that selection acting on organellar genes is relaxed following functional gene transfer to the nucleus. Examining how this relaxation influences the rate and extent of organellar gene degeneration would be of interest. In addition, models invoking the cellular costs of maintaining organellar genes may offer a valuable perspective for understanding the reductive evolution of plastid and mitochondrial genomes (Kelly, 2021).

### Genome editing and endosymbiotic gene transfer

The well-established plastid transformation system has made it possible to experimentally reconstruct plastid-to-nucleus gene transfer within laboratory timescales (Huang *et al*., 2003; Stegemann *et al*., 2003). In contrast, the lack of an efficient mitochondrial genome transformation method has long limited studies on mitochondria-to-nucleus gene transfer in plants. Plastid and mitochondrial genomes originated from different endosymbionts and differ substantially in architecture (Smith and Keeling, 2015), and their mechanisms and extent of DNA release into the cytosol are likely distinct. Given these differences, extrapolating the findings from plastid-to-nucleus gene transfer to a mitochondria-to-nucleus transfer scenario is likely inappropriate. Therefore, approaches that enable laboratory simulation of mitochondria-to-nucleus gene transfer are needed. Recent breakthroughs in mitochondrial genome editing hold promise for enabling systematic exploration of this process in the near future (Arimura and Nakazato, 2024). Meanwhile, research in animal systems has provided important insights into the formation of NUMTs. A recent study demonstrated that mitochondrial-targeted transcription activator-like effector nucleases (mitoTALEN) induce DNA breaks in the mitochondrial genome, leading to an increased rate of mitochondria-to-nucleus gene transfer (Wu *et al*., 2024). Furthermore, CRISPR–Cas9-mediated nuclear genome editing was shown to promote the integration of mitochondrial DNA at Cas9-target sites (Wu *et al*., 2024). These findings support the notion that elevated organellar DNA leakage and nuclear DSBs facilitate EGT. More importantly, they suggest that CRISPR–Cas9 target sites could potentially be harnessed to direct the integration of NUPTs and NUMTs in a controlled manner. If successful, this approach would provide a framework to investigate: (i) the effects of EGT on the local epigenetic landscape surrounding insertion sites; (ii) the impact of insertion sites on the stability of NUPTs and NUMTs; and (iii) the regulatory elements required for functional gene transfer.
